# Coronary Arteries Segmentation Based on the 3D Discrete Wavelet Transform and 3D Neutrosophic Transform

**DOI:** 10.1155/2015/798303

**Published:** 2015-01-14

**Authors:** Shuo-Tsung Chen, Tzung-Dau Wang, Wen-Jeng Lee, Tsai-Wei Huang, Pei-Kai Hung, Cheng-Yu Wei, Chung-Ming Chen, Woon-Man Kung

**Affiliations:** ^1^Institute of Biomedical Engineering, National Taiwan University, Taipei 10617, Taiwan; ^2^Department of Applied Mathematics, Tunghai University, Taichung 40704, Taiwan; ^3^Sustainability Research Center, Tunghai University, Taichung 40704, Taiwan; ^4^Cardiovascular Center and Division of Cardiology, Department of Internal Medicine, National Taiwan University Hospital, Taipei 10048, Taiwan; ^5^Department of Medical Imaging, National Taiwan University Hospital, Taipei 10048, Taiwan; ^6^Department of Nursing, College of Medicine and Nursing, Hungkuang University, Taichung 43302, Taiwan; ^7^Department of Exercise and Health Promotion, College of Education, Chinese Culture University, Taipei 11114, Taiwan; ^8^Department of Neurology, Chang Bing Show Chwan Memorial Hospital, Changhua 50544, Taiwan; ^9^Department of Neurosurgery, Lo-Hsu Foundation, Lotung Poh-Ai Hospital, Luodong, Yilan 26546, Taiwan

## Abstract

*Purpose*. Most applications in the field of medical image processing require precise estimation. To improve the accuracy of segmentation, this study aimed to propose a novel segmentation method for coronary arteries to allow for the automatic and accurate detection of coronary pathologies. *Methods*. The proposed segmentation method included 2 parts. First, 3D region growing was applied to give the initial segmentation of coronary arteries. Next, the location of vessel information, HHH subband coefficients of the 3D DWT, was detected by the proposed vessel-texture discrimination algorithm. Based on the initial segmentation, 3D DWT integrated with the 3D neutrosophic transformation could accurately detect the coronary arteries. *Results*. Each subbranch of the segmented coronary arteries was segmented correctly by the proposed method. The obtained results are compared with those ground truth values obtained from the commercial software from GE Healthcare and the level-set method proposed by Yang et al., 2007. Results indicate that the proposed method is better in terms of efficiency analyzed. *Conclusion*. Based on the initial segmentation of coronary arteries obtained from 3D region growing, one-level 3D DWT and 3D neutrosophic transformation can be applied to detect coronary pathologies accurately.

## 1. Introduction

Efficient and automatic image segmentation methods are useful for the isolation and visualization of vessels in computed tomographic angiography (CTA). There are many proposed methods for the segmentation of vessels [1–14]. A vessel filter [1] can be used to enhance tubular structure; however, it cannot address the problem of the image force and veins, which can lead to a narrowed or broken segmentation of vessels. Parametric shape models [2–5] do not directly allow for the detection of topological changes, and they usually obtain a seriously narrowed segmentation in the neighborhood of a branch point in the vessel. Level-set approaches [6–13] are computationally expensive. They also suffer from leakage at places where the intensity gradients of the edges are relatively weak and are very sensitive to the placement of the initial contour of the propagating front. Metz et al. [14] used the minimum cost path of the specified start and end points in vessel to detect the coronary arteries centerline. This is not an automatic method; detecting branches is difficult. Friman [15] proposed multiple hypothesis template tracking, which follows the direction of centerline obtained in advance. However, it is difficult to detect small branches and vessels by using this method.

In this study, we propose a new method for automatically and correctly segmenting coronary arteries from CTA data sets. In image preprocessing, we detected the aorta automatically by using methods proposed in the literature [5, 16]. The proposed coronary arteries segmentation method is summarized as follows. First of all, we automatically obtained the seed point of a 3D region growing by the difference between the two adjacent slices due to the small changes of the aorta between two adjacent slices. Next, 3D region growing was applied to initially search for the probable location of coronary arteries, which was then dilated by 3 voxels. Based on the dilation of the probable location, we detected the coronary arteries accurately by applying the 3D discrete wavelet transformation (DWT) and 3D neutrosophic transformation to the CTA volume. The location of vessel information, in HHH subband coefficients, was detected by the proposed vessel-texture discrimination algorithm. Accordingly, HHH subband coefficients were used, which were characterized and classified by *α*-means operation and *K*-means clustering. Finally, the proposed method was tested on several CTA data sets, and the experimental results indicated that the proposed method had a good performance.

The rest of this study is organized as follows.  Section 2 reviews some preliminaries, and Section 3 uses 3D region growing and 3D DWT to propose a new method for segmenting coronary arteries.  Section 4 contains the experiments and discussion, and the conclusions are drawn in Section 5.

## 2. Preliminaries

In this section, we will briefly introduce the concepts of DWT and provide an overview of some fundamental mathematical concepts that are used in this study.

### 2.1. Region Growing

Region growing is a simple, well-developed, region-based image segmentation technique [17]. It postulates that neighboring voxels within the same region have similar intensity values and is also classified as a voxel-based image segmentation method since it involves the selection of initial seed points. In other words, this method of segmentation examines neighboring voxels of initial seed points and determines whether neighboring voxels should be added to the region. Consequentially, the general concept of region growing is to group voxels with the same or similar intensities to one region according to the given seed points and a homogeneity criterion.

### 2.2. Discrete Wavelet Transform

Wavelet transform is obtained by a single prototype function *ψ*(*x*) which is regulated with scaling and shift parameters. To construct *ψ*(*x*), a scaling function *φ*(*x*) is determined. The discrete normalized scaling and wavelet basis functions are defined as

(1) $$\begin{array}{c} \varphi_{i,n}\left(t\right)=2^{i/2}\varphi \left(2^{i}t-n\right),\\ \psi_{i,n}\left(t\right)=2^{i/2}\psi \left(2^{i}t-n\right), \end{array}$$

where *i* and *n* are the dilation and translation parameters. Orthogonal wavelet basis functions not only provide a simple method to calculate coefficient expansion but also span *L* ^2^(*R*) in signal processing. As a result, signal *S*(*t*) ∈ *L* ^2^(*R*) can be expressed as a series expansion of orthogonal scaling functions and wavelets. More specifically,

(2) $$\begin{array}{c} S\left(t\right)=\underset{l}{\sum }c_{j_{0}}\left(l\right)\varphi_{j_{0},k}\left(t\right)+\underset{k}{\sum }\overset{\infty }{\underset{j=j_{0}}{\sum }}d_{j}\left(k\right)\psi_{j,k}\left(t\right), \end{array}$$

where *c*_*j*_(*l*) = ∫_*R*_ *S*(*t*)*φ*_*j*,*l*_(*t*)*dt* and *d*_*j*_(*k*) = ∫_*R*_ *S*(*t*)*ψ*_*j*,*k*_(*t*)*dt* are the low-pass and high-pass coefficients, respectively; *j*_0_ is an integer to define an interval on which *S*(*t*) is a piecewise constant. The two-scale equations for scaling and wavelet basis function are given as follows:

(3) $$\begin{array}{c} \varphi \left(t\right)=\sqrt{2}\underset{m\in Z}{\sum }h_{m}\varphi \left(2t-m\right), \end{array}$$

(4) $$\begin{array}{c} \psi \left(t\right)=\sqrt{2}\underset{m\in Z}{\sum }g_{m}\varphi \left(2t-m\right), \end{array}$$

where *g*_*m*_ = (−1)^*m*^ *h*_1−*m*_. The coefficient *h*_*m*_ in (3) has to meet several conditions for the set of the wavelet basis function to be unique and orthonormal and have a certain degree of regularity.

**Figure 1:**
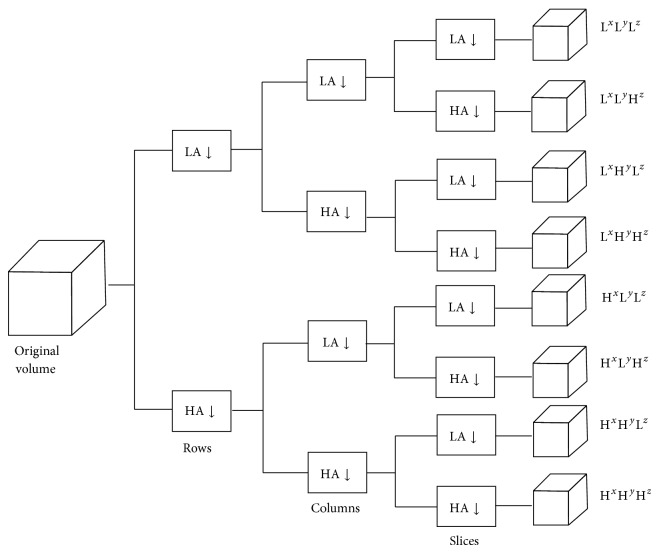
The structure of applying 3D DWT to a volume.

The coefficients *h*_*m*_ and *g*_*m*_ play a very crucial role in a given DWT. Performing the wavelet transformation does not require the explicit forms of *φ*(*t*) and *ψ*(*t*) but only depends on *h*_*m*_ and *g*_*m*_. The final output of the wavelet decomposition includes a set of *j*-level wavelet coefficients. One method to implement DWT is to use a filter bank that provides perfect reconstruction. DWT involves local analysis of frequency in space and time domains, and it provides multiscale image details step by step. If the scale becomes smaller, every part becomes more accurate, and ultimately all imaging details can be focalized accurately. If DWT is applied to a volume, it will produce the highest-frequency, middle-frequency, and lowest-frequency parts.  Figure 1 shows the results of applying 3D DWT to a volume, which includes eight parts: LLL, LLH, LHL, LHH, HLL, HLH, HHL, and HHH. The lowest-frequency and highest-frequency parts are LLL and HHH, respectively [16, 17].

### 2.3. *K*-Means Clustering

*K*-means clustering is a method of cluster analysis which aims to partition *n* observations into *k* clusters in which each observation belongs to the cluster with the nearest mean. Given a set of observations (*x*_1_, *x*_2_,…, *x*_*n*_), where each observation is a *d*-dimensional real vector, *k*-means clustering aims to partition the *n* observations into *k*  (*k* ≤ *n*) sets {*s*_1_, *s*_2_,…, *s*_*k*_} so as to minimize the within-cluster sum of squares:

(5) $$\begin{array}{c} \arg\;\min\overset{k}{\underset{i=1}{\sum }}\;\underset{x_{j}\in s_{i}}{\sum }{\left\|x_{j}-\mu_{i}\right\|}^{2}, \end{array}$$

where *μ*_*i*_ is the mean of points in *s*_*i*_.

## 3. The Proposed Segmentation Method

In order to segment coronary arteries accurately from CTA data sets, 3D region growing was initially applied to search for the probable location of the coronary arteries. Next, we used the 3D DWT and 3D neutrosophic transformation to accurately detect the coronary arteries.

### 3.1. Initial Segmentation of Coronary Arteries

This section discusses the initial segmentation of coronary arteries using 3D region growing. In image preprocessing, we found the aorta automatically by using methods proposed in the literature [5, 16]. The selection of the seed point of 3D region growing was initially made to check which slice began the information of the coronary arteries. Due to the small changes in aorta area between the two adjacent slices, we automatically obtained the seed points by the difference between the two adjacent slices.

**Figure 2:**
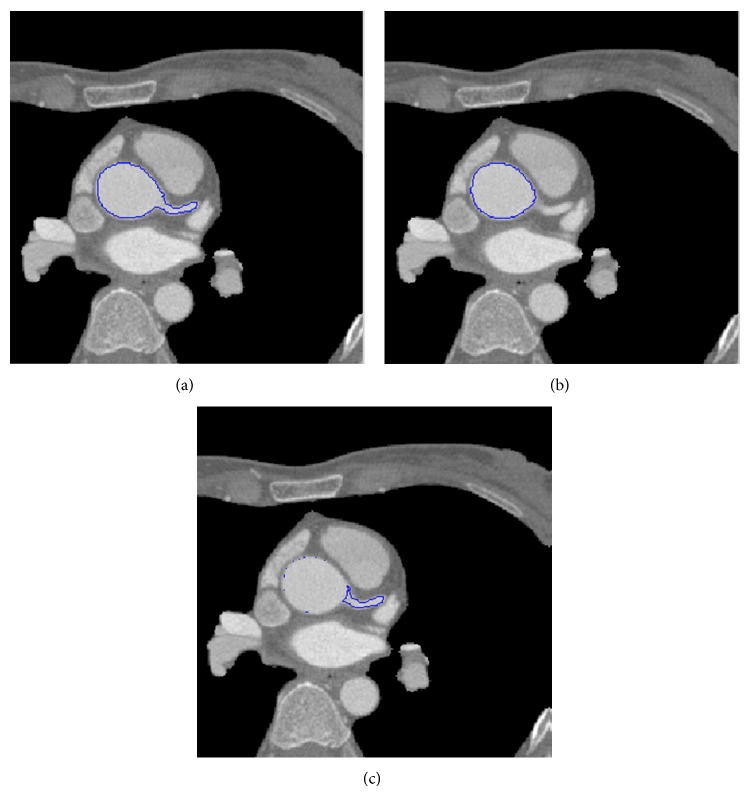
The 3D region growing seed points in (c) were automatically obtained by using the difference between the two areas bounded by the blue lines in adjacent slices (a) and (b).

As shown in Figure 2, we use the difference between the two adjacent slices (a) and (b) to automatically obtain the seed points bounded by the blue line in (c) which indicates the boundary of coronary arteries. The boundary of coronary arteries denotes the high-frequency subband *d*_*j*_(*k*) in (2) when comparing vessel lumen and background.

Since coronary arteries do not exhibit abrupt intensity changes along their centerline [4], a rough tubular mask of coronary arteries can be easily constructed by 3D region growing. We chose a 26-connected neighborhood for our adjacent pixel relationship, and then the 3D region growing method was applied with a set of prespecified seed voxel(s) and grown from these seeds by merging neighboring voxels whose properties were most similar to the premerged region. The homogeneity criterion was defined as the difference between the intensity of the candidate voxel and the average intensity of the premerged region. The selection of the seed point was initially intended to check which slice began the information of the coronary arteries. Next, the homogeneity criterion was applied to group voxels with the same or similar intensities into one region. If the homogeneity criterion was satisfied, the candidate voxel was merged with the premerged region. The process was repeated until no more voxels were assigned to the region, and then the number of all merged voxels was calculated. In order to avoid leakage, the total number of merged voxels was limited to 12000; otherwise, 3D region growing was restarted by automatically using an improved homogeneity criterion. Finally, the initial segmentation of the coronary arteries in a volume was completed.

### 3.2. Vessel-Texture Discrimination

According to Parseval's theorem, the energy in a signal *S*(*t*) is given as follows [16, 17]:

(6) $$\begin{array}{c} \int {\left|S(t)\right|}^{2}dt=\overset{\infty }{\underset{l=-\infty }{\sum }}{\left|c(l)\right|}^{2}+\overset{\infty }{\underset{j=0}{\sum }}\;\overset{\infty }{\underset{k=-\infty }{\sum }}{\left|d_{j}(k)\right|}^{2}. \end{array}$$

This equation implies that the energy of a signal is the summation of low-frequency and high-frequency coefficients. DWT is a good analytic tool for image texture analysis or line-based patterns [18–21]. Since a vessel is a type of 3D line-based pattern in CT volume, we used *l*_2_-norm⁡ of these wavelet coefficients to find the energy of line-based patterns. *l*_2_-norm⁡ was defined as

(7) $$\begin{array}{c} E\left(C\right)={\left\|C\right\|}_{2}=\overset{p}{\underset{i=1}{\sum }}{\left|c_{i}\right|}^{2}, \end{array}$$

where the vector *C* = [*c*_*i*_]_1×*p*_ was the wavelet coefficients of a frequency channel. The searching algorithm is summarized as follows.

**Algorithm 1 .**

(1) Transform a given vessel volume into frequency channels by a specified number of decomposition levels. We usually set the number to one in the first search.

(2) Use (7) to calculate the average *l*_2_-norm⁡ of each channel and maximum of *E*(*C*) for the vessel volume.

(3) If the maximum of *E*(*C*) was significantly greater than another channel's *E*(*C*), the search was stopped. Otherwise, the number of decomposition levels was increased followed by a repeat of step 1.

By using the above algorithm, we observed that the most significant information of the vessel texture often appeared in the high frequency channels. Thus, we used the subband HHH to detect vessels in this study.

### 3.3. Accurate Detection of Coronary Arteries

The initial segmentation of coronary arteries in a volume was completed by 3D region growing as described in Section 3.1. Since region growing is a simple region-based image segmentation method, it was only used to search for the initial location of the coronary arteries. We then accurately detected the coronary arteries by applying DWT to each slice in the volume, as described in this subsection.

First, the initial location of the coronary arteries obtained from 3D region growing was dilated by 3 voxels. Next, we used the Haar wavelet bases in (1) to transform the host images into the orthogonal DWT domain by one-level decomposition. Only HHH subbands were employed for further processes, because most of the information on the coronary arteries and boundaries were in the HHH subbands. We calculated the mean energy using coefficients of HHH subbands in a local window *w* as follows:

(8) $$\begin{array}{c} \bar{\text{HHH}}\left(i,j,k\right)=\frac{1}{w\times w\times w}\overset{r}{\underset{l=i}{\sum }}\;\overset{s}{\underset{m=j}{\sum }}\;\overset{t}{\underset{n=k}{\sum }}\text{HHH}\left(l,m,n\right), \end{array}$$

where

(9) $$\begin{array}{c} r=\text{round}\left(i+\frac{w}{2}\right),\\ s=\text{round}\left(j+\frac{w}{2}\right),\\ t=\text{round}\left(k+\frac{w}{2}\right). \end{array}$$

Next, the subbands HHH were characterized by 3 membership sets *T*, *F*, and *U*. Consider

(10) $$\begin{array}{c} T_{\text{HHH}}\left(i,j,k\right)=\frac{\bar{\text{HHH}}\left(i,j,k\right)-{\bar{\text{HHH}}}_{\min}}{{\bar{\text{HHH}}}_{\max}-{\bar{\text{HHH}}}_{\min}},\\ F_{\text{HHH}}\left(i,j,k\right)=1-T_{\text{HHH}}\left(i,j,k\right),\\ U_{\text{HHH}}\left(i,j,k\right)=\frac{\delta \left(i,j,k\right)-\delta_{\min}}{\delta_{\max}-\delta_{\min}}, \end{array}$$

where

(11) $$\begin{array}{c} {\bar{\text{HHH}}}_{\min}=\min\left\{\bar{\text{HHH}}\left(i,j,k\right)\right\},\\ {\bar{\text{HHH}}}_{\max}=\max\left\{\bar{\text{HHH}}\left(i,j,k\right)\right\},\\ \delta \left(i,j,k\right)=\left|\text{HHH}\left(i,j,k\right)-\bar{\text{HHH}}\left(i,j,k\right)\right|,\\ \delta_{\min}=\min\left\{\delta \left(i,j,k\right)\right\},\\ \delta_{\max}=\max\left\{\delta \left(i,j,k\right)\right\}. \end{array}$$

That is, a pixel could be represented as a neutrosophic domain *P*(*t*, *f*, *u*) which means the pixel is *t*% true, *f*% false, and *u*% uncertain, where *t* varies in *T*, *f* varies in *F*, and *u* varies in *U*. In order to reduce the uncertainty *u*%, *α*-means operation was employed as follows:

(12) $$\begin{array}{c} \overset{-}{T}\left(\alpha \right)=\left\{\begin{array}{cc} T, & \text{if}\;\;U<\alpha ,\\ {\overset{-}{T}}_{\alpha }, & \text{if}\;\;U\geq \alpha , \end{array}\right.\\ \overset{-}{F}\left(\alpha \right)=\left\{\begin{array}{cc} F, & \text{if}\;\;U<\alpha ,\\ {\overset{-}{F}}_{\alpha }, & \text{if}\;\;U\geq \alpha , \end{array}\right.\\ {\overset{-}{U}}_{\alpha }\left(i,j,k\right)=\frac{{\overset{-}{\delta }}_{T}\left(i,j,k\right)-{\overset{-}{\delta }}_{T_{\min}}}{{\overset{-}{\delta }}_{T_{\max}}-{\overset{-}{\delta }}_{T_{\min}}}, \end{array}$$

where the parameter *α* is a positive number and

(13) $$\begin{array}{c} {\bar{T}}_{\alpha }\left(i,j,k\right)=\frac{1}{w\times w\times w}\overset{r}{\underset{l=i}{\sum }}\;\overset{s}{\underset{m=j}{\sum }}\;\overset{t}{\underset{n=k}{\sum }}T\left(l,m,n\right),\\ {\bar{F}}_{\alpha }\left(i,j,k\right)=\frac{1}{w\times w\times w}\overset{r}{\underset{l=i}{\sum }}\;\overset{s}{\underset{m=j}{\sum }}\;\overset{t}{\underset{n=k}{\sum }}F\left(l,m,n\right),\\ {\bar{\delta }}_{T}\left(i,j,k\right)=\left|\bar{T}\left(i,j,k\right)-\bar{\bar{T}}\left(i,j,k\right)\right|,\\ \bar{\bar{T}}\left(i,j,k\right)=\frac{1}{w\times w\times w}\overset{r}{\underset{l=i}{\sum }}\;\overset{s}{\underset{m=j}{\sum }}\;\overset{t}{\underset{n=k}{\sum }}\bar{T}\left(l,m,n\right). \end{array}$$

The inverse DWT was then applied to obtain a new volume which possessed the true subset. Finally, we applied *K*-means clustering (*K* = 3) in (2) to differentiate vessel lumen, vessel boundary (true subset), and background. The true subset *T* was retained, respectively.

## 4. Experiments and Discussion

To test the proposed method, CTA volumes obtained from a CT system were segmented for coronary arteries. The slice thickness was 0.625 mm and the volume was 512^*^512^*^(·) in different data sets. The window size *w* was set to 3 which was enough to capture the local texture characteristics. The parameter *α* was set to 0.2. We tested 20 data sets, most of which were segmented successfully except for a few small branches that were lost in 2 of the data sets due to the local failure in region growing. To evaluate the performance of our segmented coronary arteries, we compared our results with that obtained from the ground truth values obtained from the commercial software from GE Healthcare and the level-set method. We used the overlapping metric (OM) and Hausdorff distance (*d*_*H*_) to analyze the efficiency of each method.

### 4.1. The Segmenting Efficiency of 2D Imaging

**Figure 3:**
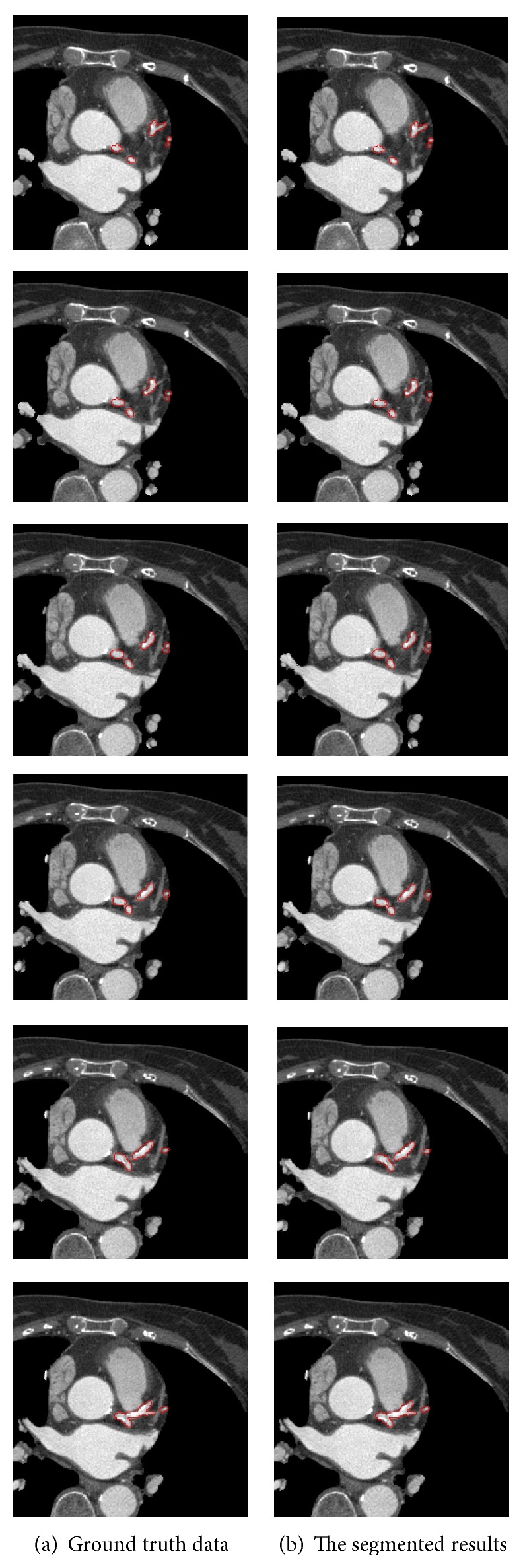
Comparison of the 2D segmentation (b) with respect to the ground truth data (a).

Figure 3(b) shows the results of 6 slices in 1 CTA volume obtained using the proposed method. The areas bounded by the red line are coronary arteries. The segmenting efficiency was compared with the manually delineated ground truth data *N*_*R*_ in Figure 3(a) by using OM which was defined as

(14) $$\begin{array}{c} \text{OM}=2\left(\frac{N_{T}\cap N_{R}}{N_{T}+N_{R}}\right), \end{array}$$

where *N*_*T*_ indicates the pixels/voxels of the segmented coronary arteries. The OM was close to 1 when the segmentation was well matched to the reference ground truth and approached zero when the results had no similarity to the reference.

In the 6 slices in Figure 3(b), the segmentation results showed that the proposed method detected coronary arteries accurately. As shown in Table 1, the average OM of the proposed method was 0.96.

### 4.2. The Segmenting Efficiency on a 3D Volume

**Figure 4:**
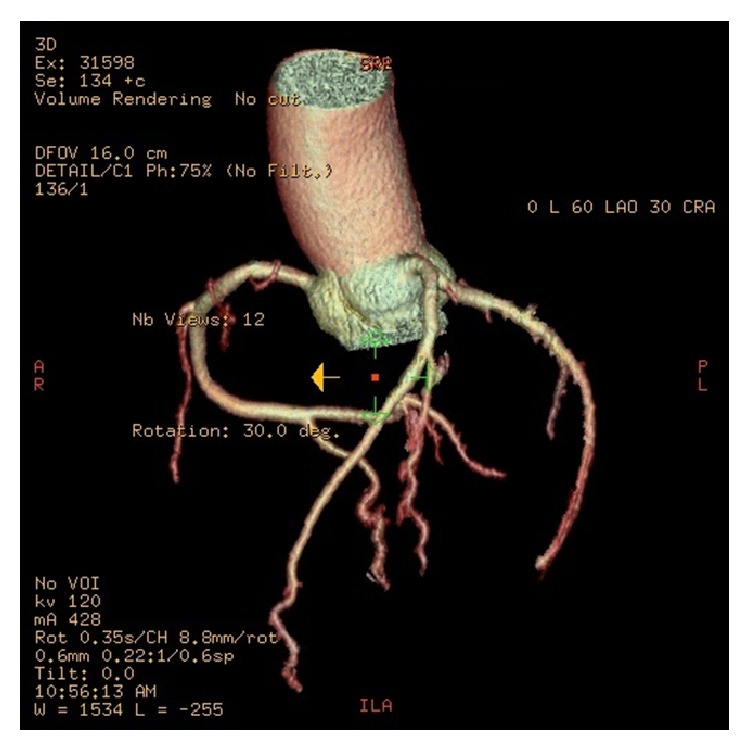
The 3D coronary arteries manually edited by a radiologist using an AW workstation (GE Healthcare, Wisconsin, USA).

**Figure 5:**
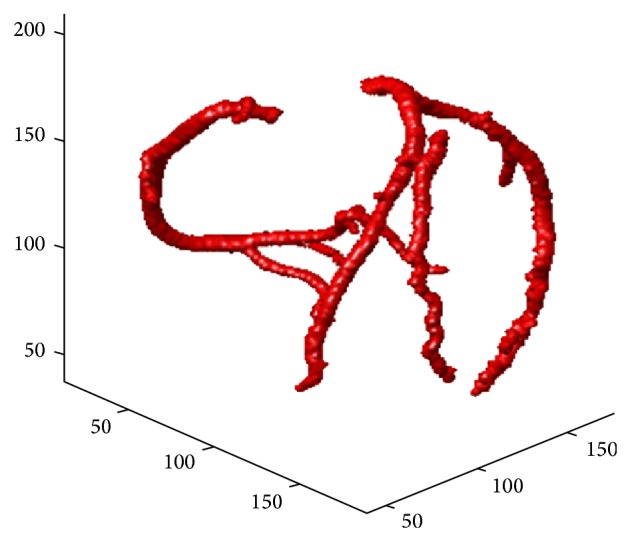
The 3D coronary arteries in the CTA volume obtained from the proposed method.

The first focus of the comparison was the correctness of the 4 main branches: the right coronary artery (RCA), the left anterior descending artery (LAD), the circumflex (CRX), and the first diagonal artery (DA). Figures 4 and 5 show the detected coronary arteries in the CTA volume obtained from the GE Healthcare and the proposed method, and the proposed method segmented these 4 main branches correctly compared to the 4 main branches in Figure 5.

**Figure 6:**
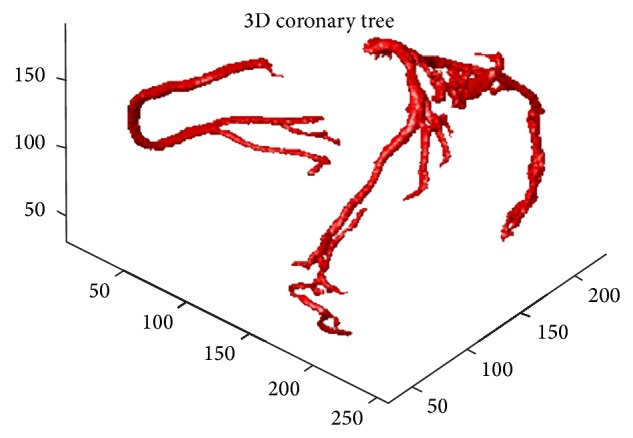
The 3D coronary arteries in the CTA volume obtained from the level-set approach.

Another test of performance is the correctness of the remaining branches. Due to the multiresolution of the DWT, each subbranch in Figures 3 and 5 was correctly segmented. Furthermore, the coronary arteries obtained from the proposed method were much better than those obtained from the level-set method, which had several leakages as seen in Figure 6.

In addition to the OM, the difference between the segmented vessel surface and the manually delineated ground truth data was measured by the Hausdorff distance [22] which was defined as follows:

(15) $$\begin{array}{c} d_{H}\left(X,Y\right)=\max\left\{\underset{x\in X}{\sup}\;\underset{y\in Y}{\inf}\;d\left(x,y\right),\underset{y\in Y}{\sup}\;\underset{x\in X}{\inf}\;d\left(x,y\right)\right\}, \end{array}$$

where *X* and *Y* are the vertices of the mesh surfaces of the arteries corresponding to the segmentation results and the ground truth and *d*(*x*, *y*) measure the Euclidean distance between points *x* and *y* belonging to vertices *X* and *Y*.  Table 2 lists the mean OM and mean Hausdorff distance for the proposed method and the method of Yang et al. [11]. The results show that the proposed method was much better than that of Yang et al. [11] in terms of both OM and Hausdorff distance.

### 4.3. Diameter Measurement

**Figure 7:**
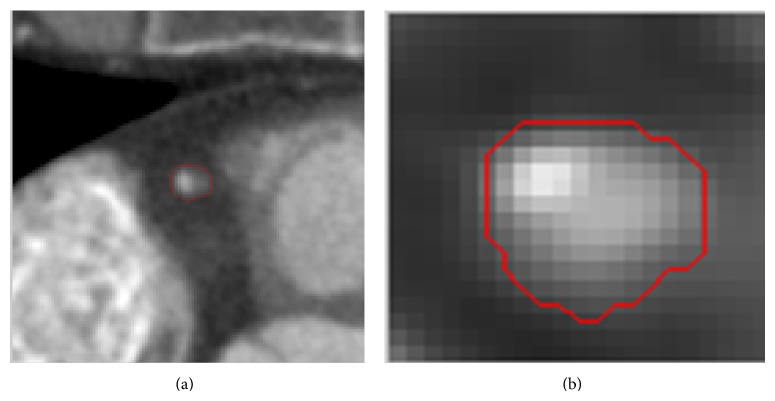
(a) The cross section of the segmented coronary arteries. (b) Magnified view of (a).

In this subsection, we computed the diameters of segmented coronary arteries using the proposed method and the method of Yang et al. Many efficient algorithms have been proposed to extract the tube centerline. We applied the algorithm proposed by Lee et al. [23] to extract the centerline of the segmented coronary arteries. Using these extracted centerlines, we obtained the cross sections of the segmented coronary arteries, as shown in Figure 7. By computing the area *A* of each cross section, the diameter *r* was estimated as follows:

(16) $$\begin{array}{c} r\approx \sqrt{\frac{A}{\pi }}. \end{array}$$

Table 3 shows the estimated diameter of the same cross section for the proposed method and the method of Yang et al. [11]. The diameter of the proposed method was closer to the diameter in ground truth data at the same cross section than to that obtained from the method of Yang et al.

### 4.4. Experimental Environment and Execution Time

The proposed method was implemented in MATLAB (R2011a) on a standard specification PC with a 3.2 GHz CPU and 12 GB RAM. The average execution time was 58 seconds to extract the entire coronary tree, compared to approximately 47 seconds for the method by Yang et al. for the same process.

## 5. Conclusions

Accurate extraction of coronary arteries is important to assess artery lesions in clinical practice. In this study, we propose a novel method to segment coronary arteries automatically. Based on the initial segmentation obtained from 3D region growing, one-level 3D DWT and 3D neutrosophic transformation were applied to detect coronary arteries accurately. The location of vessel information, in HHH subband coefficients of DWT, was successfully detected by the proposed vessel-texture discrimination algorithm. Accordingly, the HHH subband coefficients were used and characterized and classified by 3D neutrosophic transformation and *K*-means clustering. The experimental results verify the efficiency of the proposed method.

## Figures and Tables

**Table 1:** Comparison of the 2D image segmentation results.

	Yang et al. [11] method	Proposed method
Mean OM	0.68	0.96

**Table 2:** Comparison of the 3D segmentation results.

	Yang et al. [11] method	Proposed method
Mean OM	0.60	0.92
Mean *d*_*H*_(*X*, *Y*)	1.08	0.68

**Table 3:** Comparison of the cross-sectional diameter.

Ground truth	Yang et al. [11] method	Proposed method
4.7	4.4	4.8
